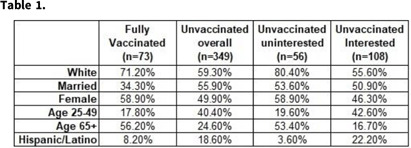# assessment of Vaccine Interest in Unvaccinated COVID-19–positive inpatients

**DOI:** 10.1017/ash.2022.136

**Published:** 2022-05-16

**Authors:** Lisa Stancill, Lauren DiBiase, Emily Sickbert-Bennett Vavalle

## Abstract

**Background:** Although vaccine hesitancy has been an issue for many years, it has become a major point of contention in the effort to mitigate the COVID-19 pandemic. In August 2021, a large academic medical facility began capturing the vaccination status of admitted COVID-19–positive patients, as well as their interest in the COVID-19 vaccine. We performed a descriptive analysis on the characteristics of unvaccinated patients who contracted COVID-19 and their interest in receiving the COVID-19 vaccine. **Methods:** Patient history and physical (H&P) notes and demographic data were collected using the internal data warehouse sourced from the electronic medical record for all SARS-COV-2–positive inpatient admissions to UNC Medical Center and UNC Chatham from August 1, 2021, to January 11, 2022. Manual chart reviews of progress notes were completed for patients whose history was not recorded in the initial H&P. Demographic data were summarized by vaccine status overall and by interest in COVID-19 vaccine among unvaccinated patients. We performed χ^2^ to determine demographic differences between the interested and uninterested unvaccinated groups. **Results:** In total, 536 patients were admitted with COVID-19 from August 1, 2021, to January 11, 2022. Of these, 15% were fully vaccinated (2 doses mRNA plus 1 dose J&J); 5.4% were partially vaccinated; 75.7% were unvaccinated; and 2.9% had an unknown vaccination status. Demographic characteristics are presented in Table 1. The most common demographics were consistent among the fully vaccinated and unvaccinated groups, with the exception of sex and age group (Table 1). For those whose interest data were available (n = 164), 34% were uninterested in receiving the COVID-19 vaccine. Importantly, race and age were statistically significantly different (*P* < .05) between the unvaccinated interested and unvaccinated uninterested groups. **Conclusions:** Even after experiencing COVID-19 firsthand and being hospitalized, some people who remain uninterested in receiving the COVID-19 vaccine. This population had a statistically higher proportion of white and older individuals than the unvaccinated interested group. Recommendations from healthcare providers might not be effective in persuading this population to be vaccinated. Instead, grassroots alternatives might be more successful. Additional analysis should be considered on whether patients who expressed interest in COVID-19 vaccine received immunization.

**Funding:** None

**Disclosures:** None

**Table:**